# Research landscape of glioma and inflammation over the past two decades

**DOI:** 10.3389/fimmu.2025.1605346

**Published:** 2025-08-13

**Authors:** Xiaoli Chen, Jiabin Wang, Shiliang Liu, Qingju Han, Haibin Zhang, Jinrong Wu

**Affiliations:** ^1^ Department of Pain Management, The First Affiliated Hospital of Harbin Medical University, Harbin, China; ^2^ Department of Neurosurgery, The First Affiliated Hospital of Harbin Medical University, Harbin, China; ^3^ Department of Neurosurgery, Xi’an Daxing Hospital, Xi’an, China; ^4^ Department of Anesthesiology, The First Affiliated Hospital of Harbin Medical University, Harbin, China

**Keywords:** bibliometrics, glioma, inflammation, VOSviewer, hotspots

## Abstract

**Introduction:**

Glioma is one of the most aggressive brain tumors, and its interaction with inflammation has become an emerging research focus. Despite increasing interest in the role of inflammation in glioma progression and therapy, a comprehensive bibliometric analysis of this field is lacking.

**Methods:**

We conducted a systematic bibliometric analysis of glioma and inflammation research using the Web of Science Core Collection (WoSCC). A total of 4,553 publications from 2005 to 2025 were analyzed for research trends, hotspots, key contributors, and emerging directions using CiteSpace and VOSviewer.

**Results:**

Our analysis revealed a significant increase in publications over the past decade. From 2005 to 2025, a total of 4,553 papers related to glioma and inflammation were published. Key contributing countries, institutions, and authors were identified, highlighting the dominance of the U.S. and China in this field. Leading institutions include MD Anderson Cancer Center and Harvard Medical School (USA) and several top Chinese universities. Keyword clustering and co-citation analysis indicated that expression, growth, and survival are major research hotspots. Highly cited papers primarily focused on molecular subtypes, immune modulation, and therapeutic resistance in glioma. ssGSEA analysis revealed that the score based on the 25-gene signature was significantly enriched in GBM and was closely associated with poor prognosis in GBM patients.

**Conclusion:**

Glioma and inflammation research have gained increasing attention, particularly in tumor immunity and microenvironment studies. This study outlines the current research landscape and trends, potentially serving as a reference for exploring future areas of investigation and collaboration.

## Introduction

Gliomas represent the most common and aggressive malignant tumors of the central nervous system, with glioblastoma (GBM) being the most lethal subtype. The highly heterogeneous biology of GBM, coupled with its resistance to conventional therapies, contributes to the dismal prognosis associated with this disease. Despite advancements in multimodal treatment approaches, including surgical resection, radiotherapy, and chemotherapy, the median survival of glioblastoma patients remains under two years, with a five-year survival rate that remains exceedingly low (1). With advancements in molecular biology and immunology, researchers have increasingly recognized that gliomas are not merely the result of uncontrolled tumor cell proliferation; rather, the complexity of their microenvironment plays a pivotal role in tumor initiation, progression, and therapeutic resistance. Consequently, a deeper exploration of the key factors influencing glioma progression, particularly inflammation-related mechanisms, is essential for the development of more effective therapeutic strategies.

Inflammation plays a central role in the initiation and progression of gliomas and is regarded as a critical factor shaping the tumor microenvironment and influencing therapeutic responses (2). The glioma microenvironment is enriched with pro-inflammatory cytokines, such as IL-1β, IL-6, and TNF-α, along with tumor-associated macrophages (TAMs) and microglia. Together, these immune components shape an immunosuppressive milieu that enables tumor cells to evade immune surveillance (3–5). Moreover, inflammatory signaling pathways, including NF-κB, JAK-STAT, and NLRP3, are aberrantly activated within the tumor microenvironment, further driving tumor cell proliferation, invasion, and the development of therapeutic resistance (6–8). In recent years, targeted therapeutic strategies against inflammation-related molecules—such as immune checkpoint inhibitors, anti-inflammatory agents, and drugs targeting tumor-associated macrophages (TAMs)—have emerged as a major focus in glioma research (9–12). Therefore, a systematic review of the current research landscape and emerging trends in glioma-related inflammation can provide deeper insights into their interplay and offer new directions for future investigations.

Bibliometric analysis is a quantitative method based on large-scale literature data, enabling the identification of academic trends, research hotspots, key authors, leading institutions, and emerging frontiers within a specific research field (13). By leveraging bibliometric tools such as CiteSpace and VOSviewer, researchers can analyze glioma and inflammation-related literature from multiple perspectives, including citation networks of high-impact papers, collaboration networks, keyword co-occurrence analysis, and burst term detection. These analyses not only help identify research hotspots but also unveil academic collaboration patterns, interdisciplinary trends, and potential future directions. Furthermore, bibliometric analysis enables the tracking of emerging technologies and research paradigms, providing researchers with a more structured academic resource to support decision-making and strategic research planning.

This study employs a bibliometric approach to systematically analyze the literature on glioma and inflammation from the past two decades, aiming to uncover global research trends, key contributors, influential publications, research hotspots, and emerging advancements in the field. Through high-frequency keyword analysis, we identified core research directions within this domain. Additionally, we examined collaboration networks among research institutions and scholars to assess academic collaboration patterns and the degree of internationalization. The findings of this study not only provide researchers with a comprehensive academic overview but also offer valuable insights for future investigations, facilitating the exploration of inflammation’s role in glioma initiation, progression, and treatment. Furthermore, this study provides a theoretical foundation for optimizing personalized therapeutic strategies.

## Materials and methods

### Data source and retrieval strategy

Relevant literature was extracted from the Web of Science Core Collection (WoSCC) database, the most trusted scientific citation database in the world (14), covering the period from 2005 to 2025. The search terms included TS = (“glioma*” OR “glioblastoma*” OR “GBM” OR “glioblastoma multiform*” OR “malignant glioma”) AND TS = (“inflammation*” OR “inflammatory”) AND DOP = (2005-01-01/2025-03-22). The wildcards * represent root word truncation of one or more other characters. For example, glioma* could refers to glioma or gliomas.

**Figure 1:**
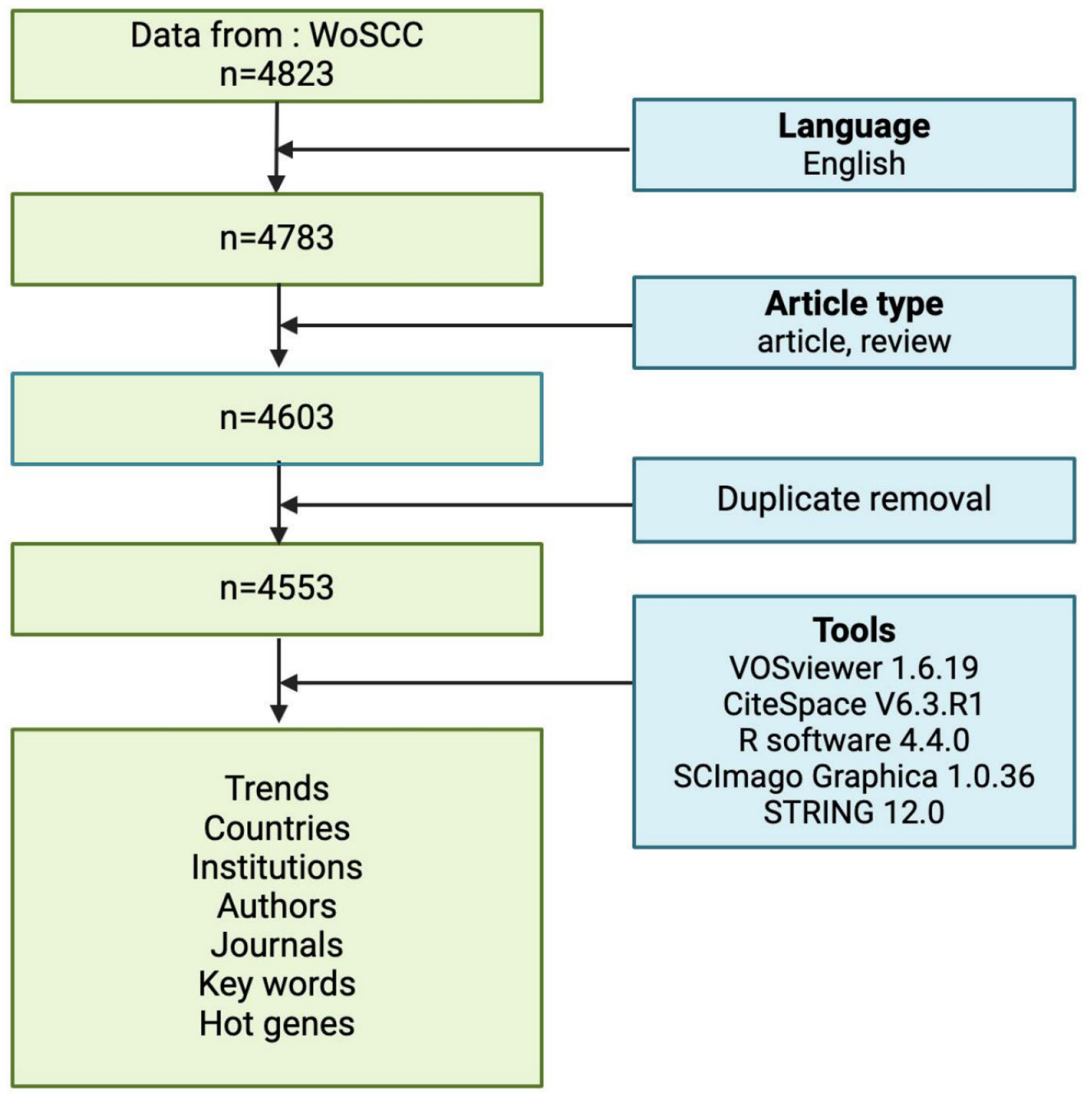
Flowchart of this study.

The inclusion criteria for the literature sources in this study were: (1) the literature search period spanned from January 1, 2005 to March 22, 2025; (2) the selected publication types of literature were “article’ and ‘review article’, and (3) the selected language was English. The bibliometric process is illustrated in flowchart **Figure 1**. Finally, 4553 records were retrieved from WoSCC.

### Data analysis and visualization

The bibliographic records meeting the above criteria were exported in plain text format, including information such as title, keywords, citation count, publication date, country/region, researchers, affiliated institutions, academic journals, impact factor (IF), and H-index. These records were then used for subsequent visualization and bibliometric analysis. The study employed Biblioshiny with R version 4.4.0 (15), SCImago Graphica (version 1.0.36) (16), CiteSpace (version 6.3.R1) (17) and VOSviewer (version 1.6.19) (18) for data importing, filtering, visualization and trend analysis. Key analyses performed included: (1) Publication trends and citation growth, (2) Co-authorship network analysis (authors, institutions, and countries), (3) Keyword co-occurrence and burst detection, (4) Co-citation analysis of influential references. Additionally, we manually extracted the top 25 hotspot genes and performed Gene Set Enrichment Analysis (GSEA) analysis and protein interaction (PPI) network construction using R language and the STRING (version12.0, https://string-db.org/) online database (19).

## Results

### Publication trends and citation analysis

**Figure 2:**
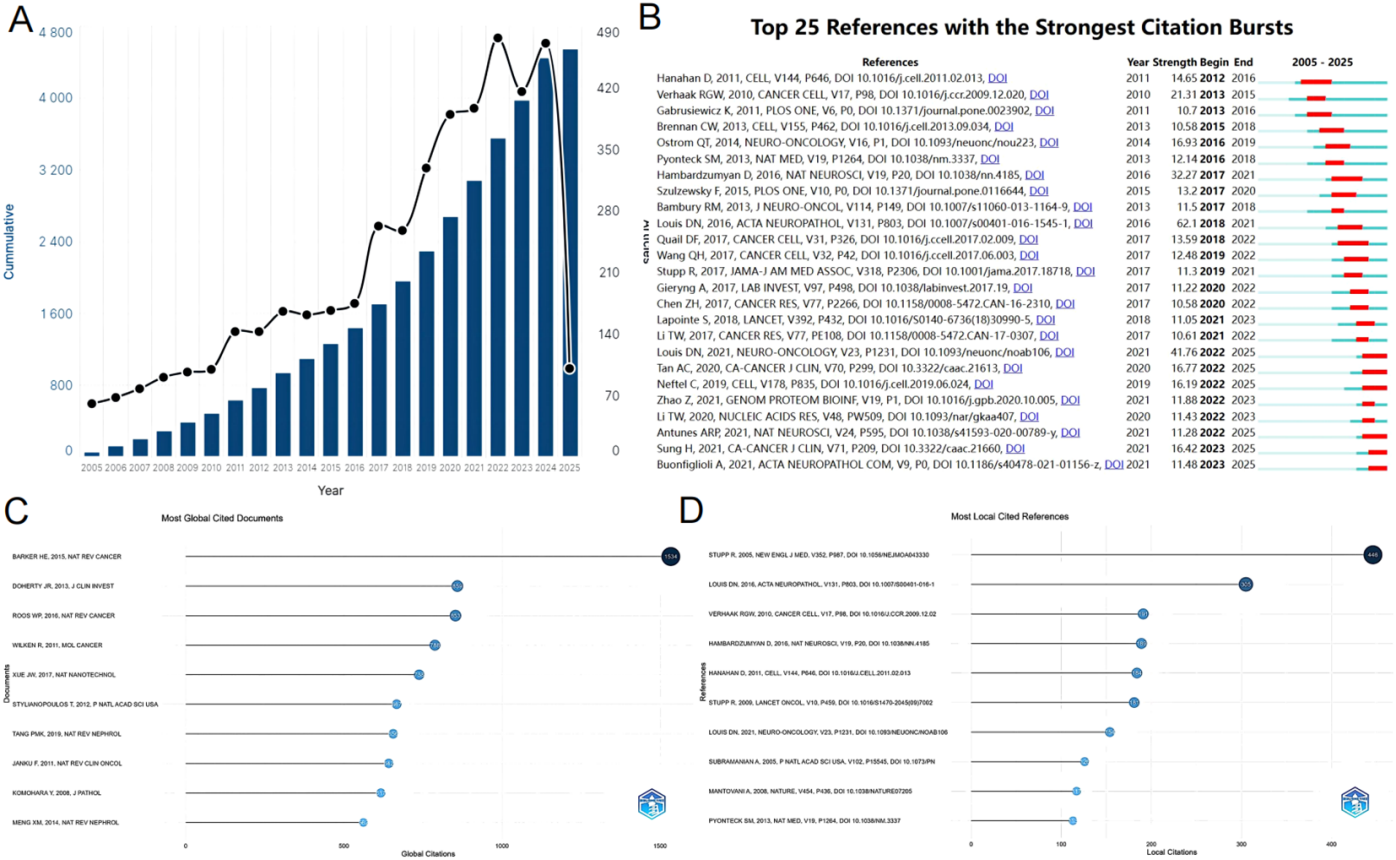
**(A)** The number of articles published annually and the cumulative number of publications. **(B)** Top 25 references with the strongest citation bursts. **(C)** Most global cited documents. **(D)** Most local cited references.

**Figure 3:**
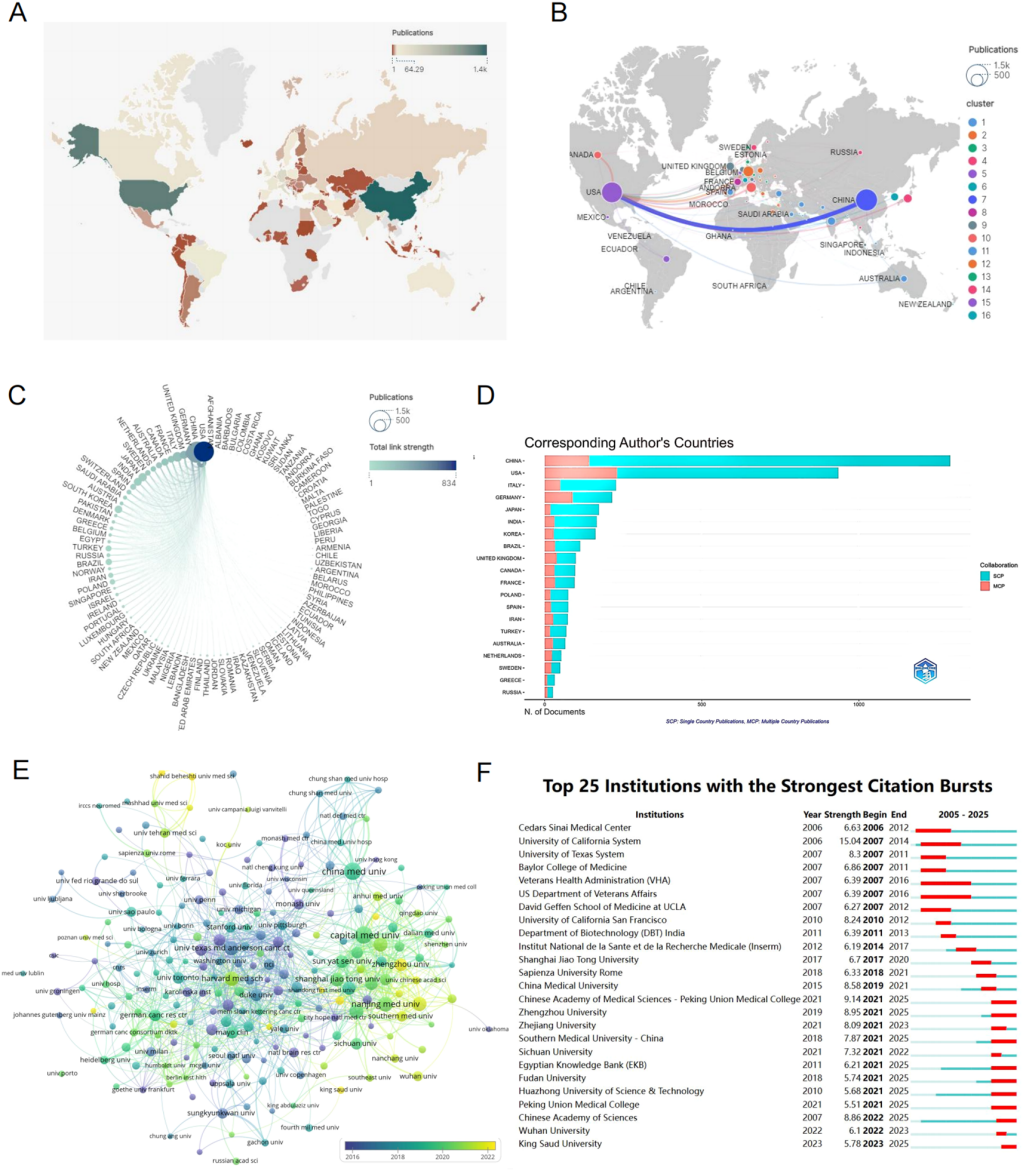
**(A)** A world map showing the distribution of publication quantities per country represented by different colors. **(B)** National and Regional Distribution of Publication Volume and Collaboration: Each node represents a country (region), with the node size indicating the number of published articles from that country (region). The lines between nodes represent the extent of collaborative activities between countries (regions). **(C)** A circular diagram of collaborative relationships between countries (regions). Each node symbolizes a country (region). The size of each node indicates the number of articles published within that country (region). The color of the nodes and the connecting lines represent the total number of interactions between the relevant nodes and other nodes. **(D)** Corresponding author country statistics chart. **(E)** Institutional clustering and temporal overlay map of publications. **(F)** Top 25 institutions with the strongest citation bursts.

From 2005 to 2025, the Web of Science Core Collection (WoSCC) has indexed a total of 4,553 studies on glioma and inflammation, including 3,600 articles and 933 reviews. Articles account for 79% of the total publications, while reviews make up nearly 21%. Analyzing the historical publication trends in this field, the number of publications has shown a gradual increase over time. Before 2010, fewer than 100 related articles were published annually. Since 2010, the annual publication count reached 100, and from 2017 onward, it surpassed 200 per year. The year 2022 recorded the highest number of publications, reaching 477. **Figure 2** presents the top 25 strongest citation bursts, with citation periods primarily concentrated between 2012 and 2025. The earliest among them is a study published by Douglas Hanahan et al. in *Cell* in 2011, titled “Hallmarks of Cancer: The Next Generation.” (20). The most strongly cited publication appeared in peer-reviewed journal *Neuro-Oncology*. Authored by David N. Louis et al. in 2021, the study is titled “The 2021 WHO Classification of Tumors of the Central Nervous System: A Summary.” (21). **Figure 3** presents the top ten most globally cited documents, covering topics such as the tumor microenvironment, lactate metabolism, DNA damage, neutrophils, macrophages, autophagy, and inflammation. The most highly cited article has been referenced 1,534 times, making it the only one to surpass 1,000 citations. This article, published in *Nature Reviews Cancer* in 2015, is a review focusing on the tumor microenvironment following radiation therapy (22). Notably, among the ten documents, only one is a research article. This study, authored by Jingwei Xue et al. and published in *Nature Nanotechnology* in 2017, is titled “Neutrophil-mediated anticancer drug delivery for suppression of postoperative malignant glioma recurrence.” (23) (**Table 1**).

**Table 1:** Top 10 most global cited articles related to Glioma and inflammation.

Title	Journal	Author	Year	Citation
The tumour microenvironment after radiotherapy: mechanisms of resistance and recurrence	*Nature Reviews Cancer*	Barker Holly E; et al (22)	2015	1534
Targeting lactate metabolism for cancer therapeutics	*Journal of Clinical Investigation*	Doherty Jason R; et al (55)	2013	859
DNA damage and the balance between survival and death in cancer biology	*Nature Reviews Cancer*	Roos Wynand P; et al (56)	2016	853
Curcumin: A review of anti-cancer properties and therapeutic activity in head and neck squamous cell carcinoma	*Molecular Cancer*	Wilken Rebecca;et al (57)	2011	788
Neutrophil-mediated anticancer drug delivery for suppression of postoperative malignant glioma recurrence	*Nature Nanotechnology*	Xue Jianwei; et al (23)	2017	738
Causes, consequences, and remedies for growth-induced solid stress in murine and human tumors	*Proceedings of the National Academy of Sciences of the United States of America*	Stylianopoulos Triantafyllos; et al (58)	2012	667
Macrophages: versatile players in renal inflammation and fibrosis	*Nature Reviews Nephrology*	Tang Patrick Ming-Kuen; et al (59)	2019	656
Autophagy as a target for anticancer therapy	*Nature Reviews Clinical Oncology*	Janku Filip; et al (60)	2011	642
Possible involvement of the M2 anti-inflammatory macrophage phenotype in growth of human gliomas	*Journal of Pathology*	Komohara Yoshihiro; et al (61)	2008	617
Inflammatory processes in renal fibrosis	*Nature Reviews Nephrology*	Meng Xiaoming; et al (62)	2014	562

### Analysis of publication countries and institutions

A total of 97 countries or regions and 261 institutions have made significant contributions to the field of glioma and inflammation research. The top ten contributing countries span three continents, including Asia (4 countries), Europe (4 countries), and North America (2 countries). Among them, China ranks first in the number of published articles, with 1,399 publications, followed by the United States with 1,250 publications. However, in terms of total citations, the United States leads with 58,412 citations, followed closely by China with 32,421 citations (**Table 2**). These findings indicate that China and the United States have made significant contributions to glioma and inflammation research. While the United States currently holds a greater influence in terms of citations, China exhibits a stronger research momentum, showing a trend of catching up and potentially surpassing in the future.

**Table 2:** Top 10 productive countries/regions related to Glioma and inflammation.

Rank	Country	N	Citations	Average article citations
1	China	1399	32421	23.17
2	USA	1250	58412	46.73
3	Germany	330	15564	47.16
4	Italy	287	9212	32.10
5	Japan	216	6673	30.89
6	United Kingdom	197	9165	46.52
7	India	193	3874	20.07
8	South Korea	177	5010	28.31
9	Canada	163	6714	41.19
10	France	146	4975	34.08

The geographical distribution map (**Figures 3A**) highlights the central roles of China and the United States in this field, with European countries also holding a considerable share. The chord diagram (**Figure 3**) further reveals that China and the United States dominate international collaborations, whereas connections among other countries remain relatively weak, suggesting that global cooperation in glioma and inflammation research still needs to be strengthened. By analyzing the corresponding authors’ national affiliations, we find that a large proportion of U.S. publications involve international collaborations. China ranks second in terms of the number of multinational collaborative publications. However, in terms of proportion, German scientists show a stronger tendency to collaborate internationally (**Figure 3**).

The institutional analysis reveals that the top ten institutions by publication volume are all based in China and the United States, including Capital Medical University (87), China Medical University (87), Nanjing Medical University (62), Central South University (59), Huazhong University of Science and Technology (57), Fudan University (57), Harvard University (56), and MD Anderson Cancer Center, University of Texas (56), among others. From a citation analysis, it is found that MD Anderson Cancer Center has the highest total citations, with 4,047 citations and an average of 72.27 citations per article. China Medical University ranks second with 2,315 total citations. Harvard University has a total of 1,983 citations, ranking fourth, but its average citation of 35.41 is the second highest. These results suggest that the two U.S. institutions have the highest citation and average citation numbers, indicating their core position in the glioma and inflammation research field. Meanwhile, Capital Medical University and China Medical University from China have the highest publication volumes, and their total citations and average citations also rank highly (**Table 3**).

**Table 3:** Top 10 productive institutions related to Glioma and inflammation.

Rank	Institutions	Country	NP	NC	Average citation
1	Capital Medical University	China	87	2062	23.70
2	China Medical University	China	87	2315	26.61
3	Nanjing Medical University	China	62	1379	22.24
4	Central South University	China	59	1024	17.36
5	Huazhong University of Science and Technology	China	57	1181	20.72
6	Fudan University	China	57	1203	21.11
7	Harvard Medical School	USA	56	1983	35.41
8	University of Texas MD Anderson Cancer Center	USA	56	4047	72.27
9	Shanghai Jiao Tong University	China	52	1255	24.13
10	Shandong University	China	50	1426	28.52

The institutional clustering diagram shows that institutions publishing on glioma and inflammation are mainly concentrated in Capital Medical University, China Medical University in China, and MD Anderson Cancer Center and Harvard University in the United States. These institutions collaborate on publications, forming a complex publication network (**Figure 3**). **Figure 3** highlights the top 25 strongest citation bursts by institution. Notably, since 2006, Cedars-Sinai Medical Center in the U.S. entered the citation burst phase. The Chinese Academy of Sciences started in this field in 2007, but it wasn’t until 2022 that it entered the burst phase.

### Journal analysis

**Figure 4:**
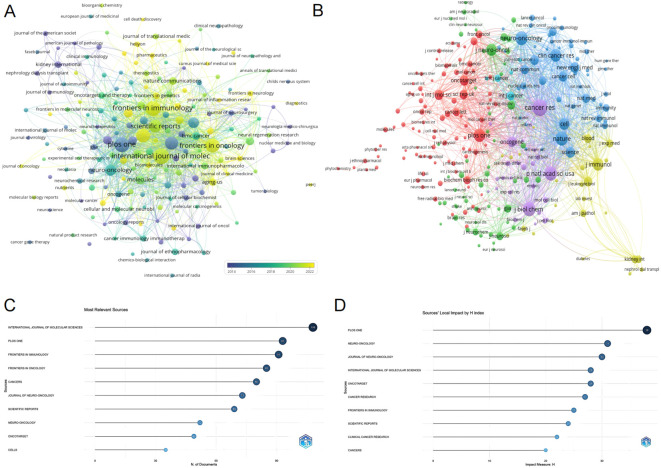
**(A)** Journal clustering and time overlay map of publications. **(B)** Journal co-citation analysis graph. **(C)** Journal publication volume chart. **(D)** Journal H-index statistics chart.

Exploring the distribution of research across different journals provides valuable insights for researchers and supports academic publishing in the field of glioma and inflammation. The journal clustering visualization (**Figure 4**) reveals the distribution of publications across various journals, with the International Journal of Molecular Sciences, PLOS ONE, and Frontiers in Immunology leading in publication volume. The citation analysis of journals (**Figure 4**) highlights the core journals in this field, with Cancer Research, Neuro-Oncology, and PLOS ONE standing out as central to the discourse on glioma and inflammation. **Figure 4** displays the top ten journals by publication volume, with the International Journal of Molecular Sciences publishing the most related studies, totaling 108 articles, ranking first. **Figure 4** shows the top ten journals by H-index, with PLOS ONE ranking first with an H-index of 38. **Table 4** presents detailed information on the top 10 journals in terms of total publication volume, total citations, average citations, and impact factor. It shows that PLOS ONE has the highest total citations (3,501), likely due to its large volume of published articles. However, Neuro-Oncology published 49 articles and has a citation total of 3,401, with an average citation of 69.41, ranking first in terms of average citations. Additionally, Neuro-Oncology has the highest impact factor among these journals, further emphasizing its significant influence in the field of glioma and inflammation. These results highlight the importance of Neuro-Oncology in the glioma and inflammation research landscape, both in terms of publication influence and citation impact.

**Table 4:** Top 10 most productive journals related to Glioma and inflammation.

Rank	Journal	NP	NC	Average citation	IF (2023)
1	International Journal of Molecular Sciences	108	2400	22.22	5.6
2	PLOS ONE	93	3501	37.65	2.9
3	Frontiers in Immunology	91	2659	29.22	5.7
4	Frontiers in Oncology	85	1202	14.14	3.5
5	Cancers	73	1474	20.19	4.5
6	Journal of Neuro-Oncology	69	2629	38.10	3.2
7	Scientific Reports	52	1338	25.73	3.8
8	Neuro-Oncology	49	3401	69.41	16.4
9	Oncotarget	35	1835	52.43	/
10	Cells	33	676	20.48	5.1

### Author analysis

**Figure 5:**
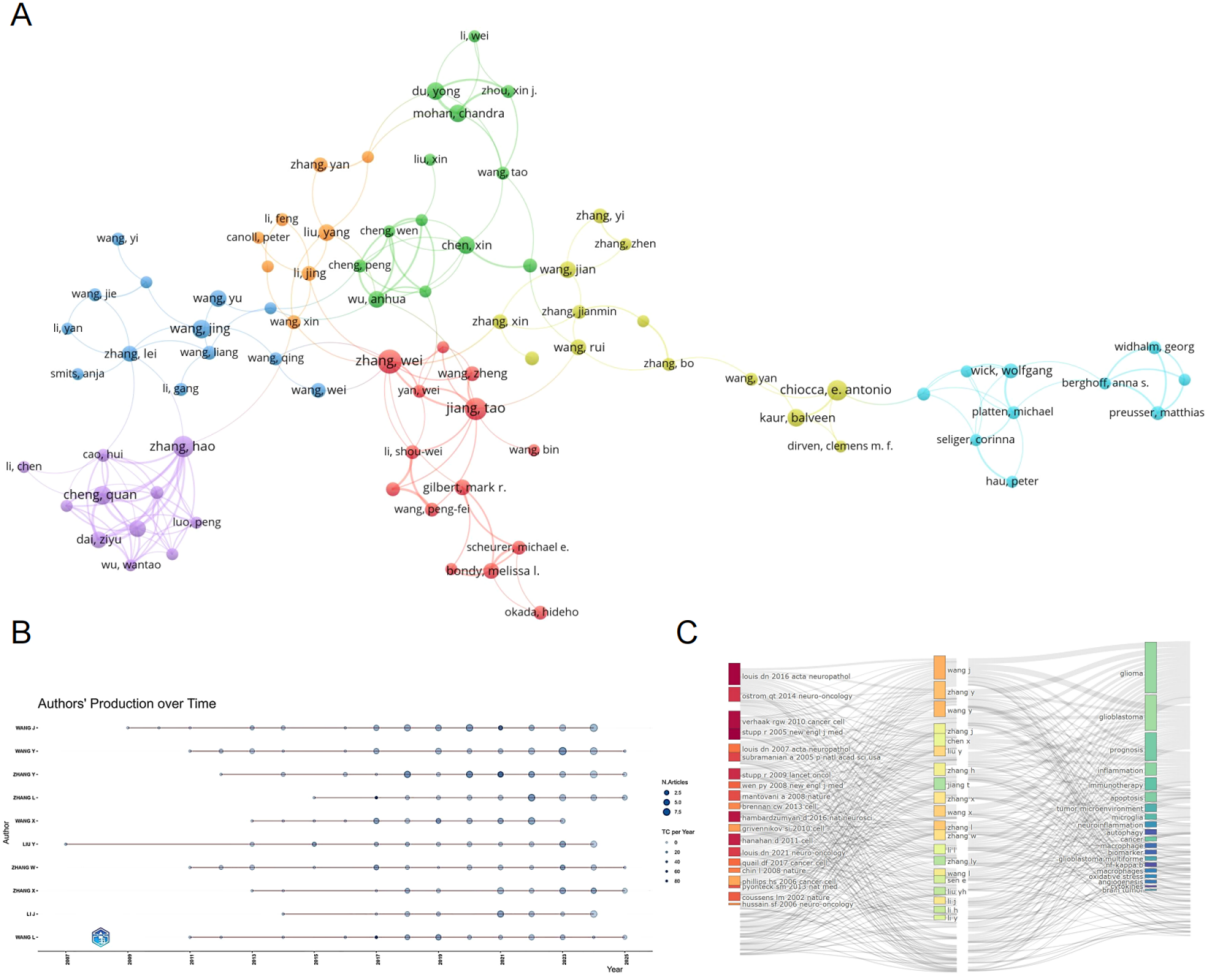
**(A)** Author collaboration network graph. **(B)** Author collaboration network graph. **(C)** Topic evolution map analysis.

We conducted an analysis of authors publishing in the field of glioma and inflammation, ranking them by total publication volume (**Table 5**). The top three authors are Zhang Wei (23 publications), Sen Ellora (21 publications), and Jiang Tao (19 publications). Two of these authors are affiliated with Beijing Tiantan Hospital, China, while one is from the National Brain Research Centre, Manesar, India. **Figure 5** presents a visualization of author collaborations, where authors are divided into 7 colors based on shared collaboration patterns. Authors with the same color have similar characteristics in their co-authorship networks. Notably, experts in glioma research such as Zhang Wei, Jiang Tao, and Wu Anhua are prominent in the visualization. **Figure 5** illustrates the publication trends of different authors over time, showing the number of articles published and their citation impact. This analysis highlights the steady increase in the publication output and citations for key authors in the field. **Figure 5** shows the relationship between authors, their highly cited articles, and keywords, indicating the areas of focus and expertise of the leading researchers in glioma and inflammation. This network analysis provides insights into the connections between prominent authors and their contributions to the field.

**Table 5:** Top 10 productive authors related to Glioma and inflammation.

Rank	Author	NP	NC	Average Citation	H-index
1	Zhang Wei	23	466	20.26	13
2	Sen Ellora	21	740	35.24	14
3	Jiang Tao	19	853	44.89	15
4	Zhang Hao	18	407	22.61	14
5	Chiocca E Antonio	16	1196	74.75	12
6	Nikolic-Paterson David J	16	1977	123.56	14
7	Kim Sun Yeou	15	365	24.33	11
8	Cheng Quan	14	361	25.79	13
9	Badie Behnam	14	882	63.00	13
10	Wang Jing	14	119	8.50	19

### Keyword analysis

**Figure 6:**
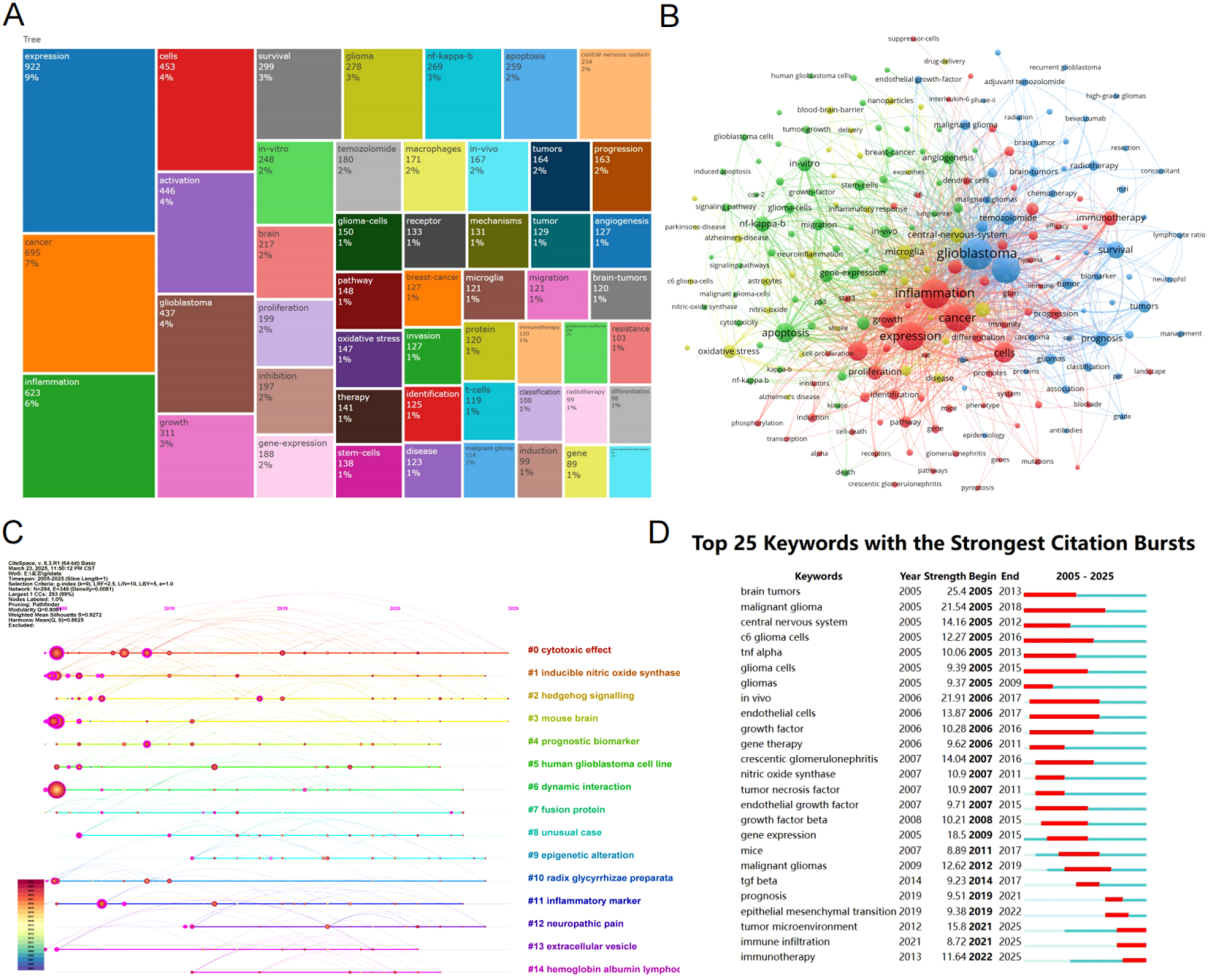
**(A)** Keyword tree map analysis. **(B)** Keyword clustering map. **(C)** Timeline graph of reference co-citation analysis. **(D)** Top 25 keywords with the strongest citation bursts.

Keywords are labels for research content, and they can reveal research hotspots and trends. We performed a treemap analysis of keywords, with different colors representing different keywords, and the size of the area indicating the frequency of keyword occurrence. The analysis shows that expression, cancer, and inflammation are the most dominant keywords (**Figure 6**). **Figure 6** presents the visualization of keyword clustering analysis, where the size of the nodes represents the frequency of keyword occurrences, and the distance between nodes indicates the strength of the connection between keywords. Keywords that are close together in distance are clustered, reflecting the primary research focus in the field of glioma and inflammation. The clusters can be roughly divided into three categories: The first group, represented by blue, includes keywords such as glioma, hypoxia, survival, and TMZ. The second group, represented by red, includes keywords like expression, inflammation, growth, and cancer. The third group, represented by green, includes keywords such as apoptosis, angiogenesis, NF-kappaB, and migration. **Figure 6** is a timeline chart, showing that research in glioma and inflammation primarily revolves around 14 key themes. We observed that keywords such as cytotoxic effect, inducible nitric oxide synthase, and hedgehog signaling have been the most frequently appearing keywords in recent years.

To further understand the emergence and duration of keywords, we used CiteSpace for a keyword burst analysis (**Figure 6**). We found that malignant glioma appeared the earliest and has had the longest burst duration. In contrast, in recent years, tumor microenvironment, immune infiltration, and immunotherapy have emerged as the most popular research topics.

### Analysis of key genes in glioma and inflammation research

**Figure 7:**
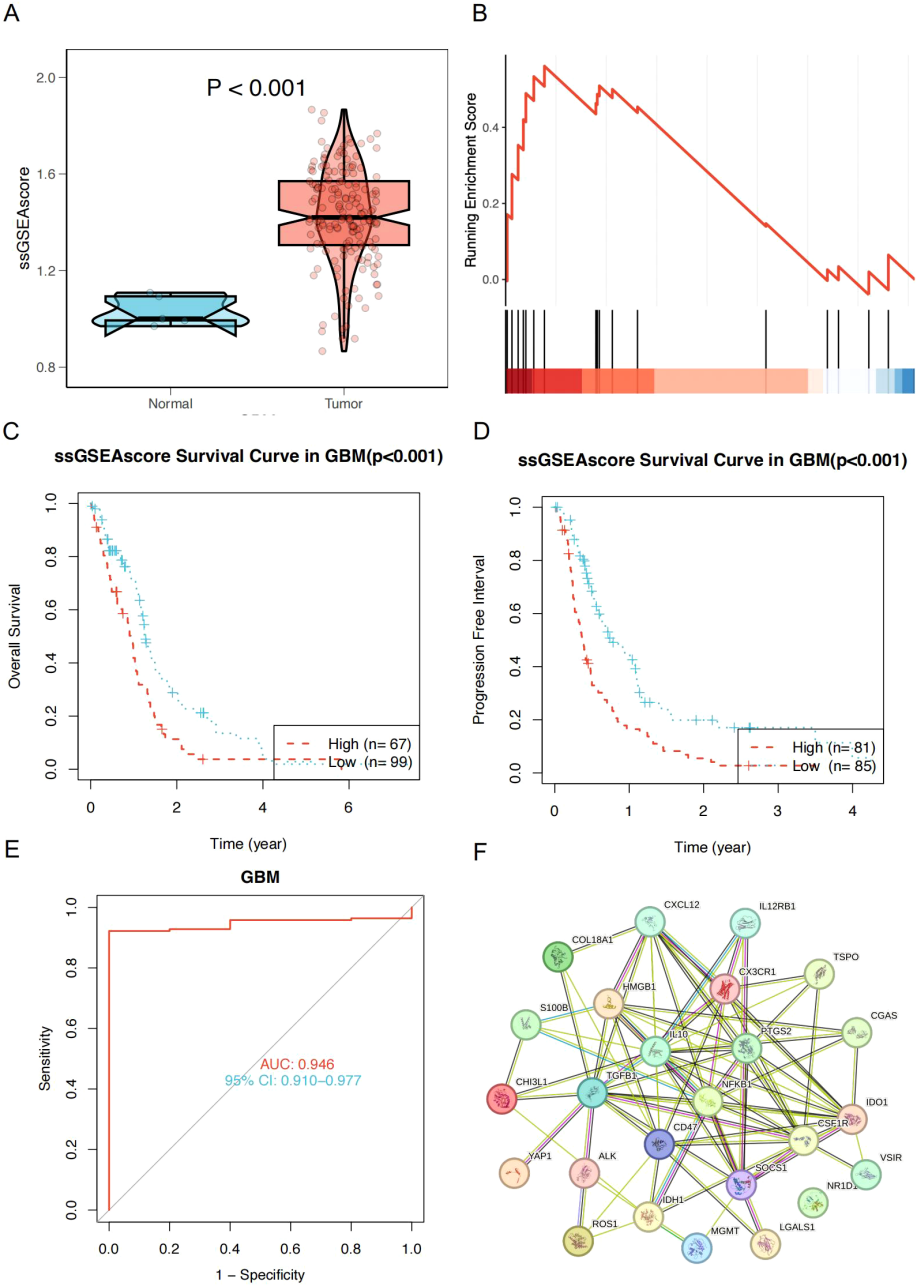
**(A)** ssGSEA scores were significantly higher in GBM tumor tissues compared to normal tissues (P < 0.001). **(B)** GSEA Analysis Plot of Top 25 Hot Genes. **(C)** Kaplan–Meier overall survival curves for GBM patients stratified by high and low ssGSEA scores (P < 0.001). **(D)** Progression-free survival curves for GBM patients stratified by high and low ssGSEA scores (P < 0.001). **(E)** Receiver operating characteristic (ROC) curve of the ssGSEA score in diagnosing GBM; AUC = 0.946, 95% CI: 0.910–0.977. **(F)** Protein-Protein Interaction (PPI) Network Diagram.

To further identify the key research genes in the field of glioma and inflammation, we ranked genes based on total citation counts and manually selected the top 25 most influential genes. To explore the roles of these genes in glioma, we performed Gene Set Enrichment Analysis (GSEA) on the gene set composed of these 25 genes using TCGA-GBM data. The results showed that the ssGSEA score of this pathway was significantly higher in GBM tumor tissues compared to normal brain tissues and was markedly enriched in GBM (**Figures 7A**). In the TCGA-GBM cohort, survival analysis based on the ssGSEA score revealed that patients in the high-score group had significantly worse overall survival (OS) and progression-free survival (PFS) than those in the low-score group (**Figures 7C**), indicating that elevated pathway activity is closely associated with poor prognosis. We further evaluated the diagnostic performance of this score in GBM using a receiver operating characteristic (ROC) curve, which yielded an AUC of 0.946 (95% CI: 0.910–0.977), demonstrating strong discriminatory power (**Figure 7**). To further clarify their functional mechanisms in GBM, we conducted a protein-protein interaction (PPI) analysis using the STRING online database (**Figure 7**). The results revealed that CSF1R, TGFBI, and IDO1 serve as key nodes in the protein interaction network, suggesting their critical roles in glioma progression and tumor-associated inflammation.

## Discussion

Bibliometric analysis, as an important research method, has been widely applied across multiple fields. By conducting quantitative analysis of publications, researchers can uncover research trends, hotspots, and academic impact within a specific domain (24). This method not only helps researchers gain a better understanding of the knowledge structure within a specific field but also offers insights into emerging trends and potential areas of interest for future research. However, bibliometric analysis has not yet been conducted in the field of glioma and inflammation. In this study, we performed a systematic literature search using the WoSCC database and, after screening, included 4,553 publications from the past two decades related to glioma and inflammation for bibliometric analysis and visualization. By systematically analyzing the research landscape in this field, we aim to reveal development trends, research hotspots, and emerging directions, enabling researchers to quickly grasp the evolution of this domain and providing valuable references for future studies.

We found that the most locally cited article is *“Radiotherapy plus concomitant and adjuvant temozolomide for glioblastoma” (446 citations)* (1). This clinical study demonstrated that combining radiotherapy with temozolomide (TMZ) after surgery extended the median survival of GBM patients from 12.1 months to 14.6 months. This research laid the foundation for the radiotherapy + TMZ regimen as the standard treatment for GBM, significantly transforming the clinical management of the disease.

While the radiotherapy + TMZ regimen has become the standard of care, its efficacy remains limited, highlighting the need for future research to explore more effective therapeutic strategies, such as combination immunotherapy and personalized precision medicine. In the citation burst analysis, we identified that the earliest highly cited paper with the strongest citation burst was published in 2010: *“Integrated genomic analysis identifies clinically relevant subtypes of glioblastoma characterized by abnormalities in PDGFRA, IDH1, EGFR, and NF1.”* This study played a pivotal role in glioblastoma classification, revealing distinct molecular subtypes based on genetic alterations in PDGFRA, IDH1, EGFR, and NF1. Its strong citation burst in the early stages suggests that molecular subtyping was a major research focus at the time, laying the groundwork for subsequent advancements in targeted therapy and personalized treatment approaches for GBM (25). This groundbreaking study pioneered the molecular subtyping of GBM, significantly advancing the application of precision medicine in GBM research (26–28).

The regional distribution map reveals that the United States and China are the leading countries in terms of publication volume in the field of glioma and inflammation research, with their output significantly higher than that of other countries. However, despite both China and the U.S. leading in publication volume, total citations and average citations per paper show that the U.S. significantly outperforms China. This disparity points to differences in academic influence and research quality between the two countries in the glioma and inflammation field. For China to further enhance its academic influence, it will need to focus more on research innovation, international collaboration, and the publication of high-quality papers, aiming to increase the global academic impact of its research.

The distribution of corresponding authors by country reveals that China has the highest number of domestic collaboration publications (single-country publications), while the number of international collaboration publications (involving multiple countries) is lower than that of the United States. This phenomenon reflects the differences in academic collaboration models between China and the U.S., as well as the collaboration trend and academic influence of different countries in the field of glioma and inflammation research. From another perspective, due to the U.S.’s leading position, scholars worldwide tend to collaborate with U.S. researchers. In the future, China can enhance international cooperation and promote the publication of high-impact papers internationally to further strengthen its global academic influence in the glioma and inflammation research field. Author analysis helps us identify the core scientists, academic networks, collaboration patterns, research hotspots, and future trends in glioma and inflammation research. Through this analysis, we found that collaboration among researchers is primarily concentrated within the same country, institution, and field. This indicates that the research relies heavily on internal collaborations within laboratories or research teams, such as close ties between large medical research centers or university laboratories. The advantage of this collaboration model is that it allows for clear division of labor and easy resource sharing. However, it may also limit the breadth of research perspectives.

Inflammatory processes play a pivotal role in a wide range of diseases and are widely recognized as key drivers—and even initiators—of pathological changes. In tumors of the central nervous system, gliomas represent the most common and aggressive type, and their initiation and progression are closely associated with chronic inflammation (29). A growing body of evidence indicates that inflammatory signaling plays a critical role in the initiation, progression, immune evasion, and therapeutic resistance of glioma (30, 31). With the advancement of high-throughput sequencing technologies, single-cell RNA sequencing (scRNA-seq) has emerged as a powerful tool for investigating the heterogeneity of the tumor microenvironment and intercellular interactions. Through single-cell transcriptomic and spatial transcriptomic analyses of glioma patient samples, murine glioma models, and organoids, researchers have uncovered how glioma cells interact with myeloid-derived cells—including tumor-associated macrophages (TAMs), tumor-associated neutrophils, microglia, and other immune cell types—to establish a highly immunosuppressive microenvironment that further promotes tumor progression and therapeutic resistance (32–35). Emerging evidence highlights the complex interplay between glioma cells and the inflammatory microenvironment. In this context, inflammation is not merely a host response to the tumor but also an active participant in shaping glioma biology. Tumor-associated macrophages (TAMs), along with cytokines such as IL-1β, IL-6, and transforming growth factor-β (TGF-β), contribute to the establishment of a highly immunosuppressive and tumor-promoting niche (36). Activation of inflammatory pathways such as NF-κB and STAT3 not only promotes glioma cell invasion and angiogenesis but also sustains the stem-like properties of glioma stem-like cells (GSCs), ultimately contributing to tumor recurrence and poor prognosis (37, 38).

Despite the detrimental role of inflammation in glioma progression, it also offers potential therapeutic opportunities. Targeting key inflammatory mediators—such as the CCL2/CCR2 axis, CSF1R, or the IL-1β signaling pathway—has demonstrated promising anti-tumor effects in preclinical models (39, 40). Similarly, reprogramming tumor-associated macrophages (TAMs) from a tumor-promoting M2-like phenotype to a tumor-suppressive M1-like phenotype has emerged as a viable strategy to overcome the immunosuppressive tumor microenvironment (41). Moreover, the combination of standard therapy with anti-inflammatory agents, such as ruxolitinib, has already entered phase II clinical trials for the treatment of glioma patients. However, due to the dual and context-dependent role of inflammation in glioma biology, translating these approaches into clinical benefit remains a significant challenge.

With the rapid advancement of artificial intelligence (AI), machine learning (ML) and deep learning (DL) techniques have been widely applied in various aspects of glioma management, including diagnosis, grading, prognosis prediction, and molecular subtype classification. In the field of imaging diagnosis, deep learning models—particularly convolutional neural networks (CNNs) based on MRI and PET images—have demonstrated the ability to perform automatic tumor segmentation, boundary delineation, and grade classification, thereby significantly improving diagnostic efficiency and accuracy (42). At the pathological diagnostic level, AI models can automatically analyze digitized histopathological slides to predict tumor grade, quantify cell density, and assess cellular atypia, thereby assisting pathologists in making more objective and consistent evaluations (43).

In this study, we manually selected the 25 most prominent genes from the most popular research, including S100B, IDO, VEGFA, MGMT, TGFBI, CGAS, CSF1R, PTGS2, HMGB1, CHI3L1, ROS1, NR1D1, IDH1, VISTA, SOCS1, NFKB1, LGALS1, CXCL12, IL12, ALK, TSPO, CD47, YAP, IL10, CX3CR1. These genes have gained significant attention in the field of glioma and inflammation. Studies have shown that VEGFA plays a critical role in promoting angiogenesis, stemness maintenance, and immune evasion in glioma. Bevacizumab has already been used in the treatment of certain glioma patients, targeting VEGFA to inhibit blood vessel formation and tumor growth (44–47). IDH1 mutations are commonly found in low-grade gliomas, but are rare in glioblastomas (GBM). These mutations are associated with a relatively better prognosis for patients with low-grade gliomas (38–40). MGMT (O-6-methylguanine-DNA methyltransferase) methylation in patients significantly enhances the efficacy of temozolomide (TMZ) treatment. Moreover, MGMT also plays a role in regulating angiogenesis and might represent a potential predictive biomarker for glioma patients’ response to radiotherapy (48–52). TGFBI (Transforming Growth Factor Beta Induced) plays a crucial role in maintaining glioma stemness and promoting tumor growth. It is involved in regulating the tumor microenvironment, cell signaling pathways, and cellular interactions that support the growth and maintenance of glioma stem cells, which are critical for the tumor’s aggressive nature and resistance to treatment (38, 53). CSF1R (Colony Stimulating Factor 1 Receptor) promotes the recruitment of immune-suppressive macrophages to the tumor site, contributing to the immunosuppressive microenvironment that facilitates glioma progression and resistance to therapies. Targeting and blocking CSF1R has emerged as a promising therapeutic strategy for glioma, as it could inhibit macrophage recruitment, reprogram the immune landscape, and potentially enhance the effectiveness of other treatments, such as immunotherapy (40, 54). However, the presence of the immunosuppressive microenvironment in glioma makes its treatment less effective. In conclusion, the research focus in the field of glioma and inflammation is gradually shifting towards tumor immunity and the tumor microenvironment, which may serve as a potential breakthrough for glioma treatment.

## Limitation

Several limitations of this study should be acknowledged. First, the analysis was confined to publications written in English. This decision was made to ensure consistency and to facilitate accurate interpretation of the bibliometric data; however, it may have resulted in the exclusion of relevant research published in other languages, introducing a potential language bias. Second, the data source was limited to the Web of Science Core Collection (WoSCC), which was selected because of its rigorous indexing standards, comprehensive citation information, and widespread use in bibliometric research. Nevertheless, this choice may have led to the omission of pertinent studies indexed in other databases, such as Scopus, PubMed, or regional repositories. While these constraints were necessary to maintain the feasibility and comparability of the analysis, they may limit the completeness and generalizability of the findings. Future studies could address these limitations by incorporating literature from multiple databases and including non-English publications to provide a more comprehensive overview of the research landscape.

## Conclusion

Overall, research on glioma and inflammation is receiving increasing attention, largely because tumor immunity and the tumor microenvironment are seen as promising solutions to the challenges in glioma treatment. This article summarizes the key papers, core journals, keywords, research institutions, and authors in the field of glioma and inflammation. In conclusion, inflammation is a double-edged sword in glioma pathogenesis. While it supports tumor growth and immune escape, it also provides potential vulnerabilities that can be exploited therapeutically. Future research should focus on deciphering the spatiotemporal dynamics of inflammatory signals in gliomas and developing precise strategies to modulate the glioma-associated inflammation for therapeutic gain.

## Data Availability

The original contributions presented in the study are included in the article/supplementary material, further inquiries can be directed to the corresponding authors.
